# RIG012 assists in the treatment of pneumonia by inhibiting the RIG-I-like receptor signaling pathway

**DOI:** 10.3389/fmed.2024.1501761

**Published:** 2024-11-01

**Authors:** Shi Zhang, Hanbing Chen, Jianfeng Xie, Lili Huang

**Affiliations:** ^1^Jiangsu Provincial Key Laboratory of Critical Care Medicine, Department of Critical Care Medicine, Zhongda Hospital, School of Medicine, Southeast University, Nanjing, China; ^2^Department of Respiratory and Critical Care Medicine, Central Hospital Affiliated to Shandong First Medical University, Jinan, China

**Keywords:** pneumonia, RIG012, RIG-I, GSVA, treatment

## Abstract

**Objective:**

Pneumonia is a common clinical condition primarily treated with antibiotics and organ support. Exploring the pathogenesis to identify therapeutic targets may aid in the adjunct treatment of pneumonia and improve survival rates.

**Methods:**

Transcriptomic data from peripheral blood of 183 pneumonia patients were analyzed using Gene Set Variation Analysis (GSVA) and univariate Cox regression analysis to identify signaling pathways associated with pneumonia mortality. A pneumonia mouse model was established via airway injection of *Klebsiella pneumoniae*, and pathway-specific blockers were administered via tail vein infusion to assess whether the identified signaling pathways impact the mortality in pneumonia.

**Results:**

The combination of GSVA and Cox analysis revealed 17 signaling pathways significantly associated with 28-day mortality in pneumonia patients (*P* < 0.05). Notably, the RIG-I-like receptor signaling pathway exhibited the highest hazard ratio of 2.501 with a 95% confidence interval of [1.223–5.114]. Infusion of RIG012 via the tail vein effectively inhibited the RIG-I-like receptor signaling pathway, significantly ameliorated lung injury in pneumonia mice, reduced pulmonary inflammatory responses, and showed a trend toward improved survival rates.

**Conclusion:**

RIG012 may represent a novel adjunctive therapeutic agent for pneumonia.

## Introduction

Pneumonia is a common clinical syndrome that severely jeopardizes public health. It accounts for ~10–20% of admissions to intensive care units (ICU). The hospital mortality rate for pneumonia patients varies between 12 and 38%, with mortality rates as high as 40–45% among patients with severe pneumonia in the ICU (1, 2). Thus, pneumonia represents a significant clinical challenge that both clinicians and specialists in critical care medicine must confront.

Currently, the treatment of pneumonia primarily relies on antibiotics and organ support therapies, with a notable lack of adjunctive treatments targeting its underlying pathogenesis (3, 4). Investigating the mechanisms associated with the prognosis of pneumonia patients may provide insights for developing adjunctive therapies, thereby enhancing the efficacy of pneumonia management and improving patient outcomes.

Utilizing algorithms such as machine learning for the secondary analysis of patient transcriptomic data offers new perspectives for understanding diseases and aids in uncovering mechanisms associated with disease progression and prognosis (5, 6). Gene Set Variation Analysis (GSVA) is an algorithm developed by Guinney et al. that transforms transcriptomic data into signaling pathway information through a machine learning framework (7). This algorithm reduces the dimensionality of high-dimensional transcriptomic data into actionable insights about signal transduction pathways, thereby facilitating further exploration of disease-related mechanisms and drug development (7, 8).

This project conducts GSVA on transcriptomic data from 183 pneumonia patients to identify signaling pathways associated with patient prognosis. Additionally, animal experiments are employed to validate the bioinformatics findings.

## Materials and methods

### The transcriptomic data of pneumonia patients

The transcriptomic data of pneumonia patients are derived from high-throughput sequencing of peripheral blood from ICU patients uploaded to the Gene Expression Omnibus (GEO) public database (accession number GSE65682) by Scicluna et al. The dataset includes information on patients diagnosed with pneumonia, encompassing 28-day survival data and high-throughput sequencing information. This dataset was chosen because it represents the largest publicly available collection of high-throughput sequencing data for pneumonia patients, along with corresponding survival information.

### GSVA

The transcriptomic data were transformed into signaling pathway data using the GSVA algorithm developed by Guinney and the publicly available GSVA R package (Bioconductor—GSVA). The principles of the algorithm and detailed procedures are referenced in the documentation provided with Guinney's R package.

### Statistical analysis

Univariate Cox analysis was performed to identify signaling pathways associated with the 28-day mortality rate in pneumonia patients, using a significance threshold of *P* < 0.05. Based on the median of the signaling pathway data, patients were categorized into high-risk and low-risk groups. Survival analysis was conducted using the log-rank test to compare the survival outcomes between the two groups. Differential analysis was carried out using one-way analysis of variance, with *P* < 0.05 considered statistically significant.

### Mouse model of pneumonia

According to previous studies on the RIG-I pathway, there is no significant association between the RIG-I pathway and sex (10–12). The study subjects consisted of male C57BL/6 mice aged 6–8 weeks. A pneumonia model was established by intratracheal injection of *Klebsiella pneumoniae* (KP) at a dosage of 60 × 10^8^ CFU/kg over a duration of 24 h. The sham-operated group received an equal volume of PBS via intratracheal injection. Each group comprised six mice, with three designated for survival analysis and the other three euthanized after 24 h to collect lung tissue for subsequent experiments.

KP: SHBCCD11105CMCC46117; Shanghai Bioresource Collection Center, Shanghai, China.

### RIG012 treatment

Two hours after intratracheal injection of *Klebsiella pneumoniae* (KP), the RIG-I-like signaling pathway-specific inhibitor RIG012 was administered via tail vein infusion at a dosage of 5 mg/kg. Based on the RIG012 reagent specifications and prior research data on RIG012 (21), we selected this dosage, and experimental evidence confirms that 5 mg/kg is indeed effective.

The treatment control group received an equivalent volume of PBS.

RIG012: HY-147124, MedChemExpress, America.

### Western blots

Based on previous studies (9–12), the stimulation and variation of the RIG-I-like signaling pathway were evaluated through measuring the pIRF3 levels in mouse lung tissue proteins. Proteins were separated using an SDS-PAGE-PVDF membrane system. The primary antibody (1:1000) was incubated at room temperature for 2 h. Following thorough rinsing of the PVDF membrane, a secondary antibody was applied for 1 h. Protein detection was performed using enhanced chemiluminescence. The antibody information is as follows:

βTubulin Abcam, rabbit mAb, #ab108342.

IRF3 Abcam, rabbit mAb, #ab68481.

pIRF3 Cell Signaling Technology, rabbit mAb, 29047.

### Evaluation of lung injury

After paraffin embedding, the right upper lobe of the lung was sectioned into 5-micron-thick slices for histopathological examination. The sections were stained with hematoxylin and eosin (H&E). Histopathological features, including edema, inflammation, hemorrhage, atelectasis, necrosis, and hyaline membrane formation, were scored on a scale from 0 to 4. The total score reflects the severity of lung injury.

### qPCR

Based on our previous experimental foundation, the pneumonia model in mice becomes relatively stable 24 h after the intratracheal injection of KP, making it suitable for further research (20). Therefore, quantitative PCR (qPCR) was employed to assess the expression levels of the pro-inflammatory cytokines interleukin 1 beta (IL1B) and tumor necrosis factor (TNF), as well as the anti-inflammatory cytokines IL10 and transforming growth factor-beta (TGFB) in lung tissue homogenates from each group of mice 24 h post-injection of KP. This analysis aimed to evaluate the impact of RIG012 on the pulmonary inflammatory response in pneumonia-induced mice. The primer information is as follows:

IL1B

F: GCCACCTTTTGACAGTGATG

R: CGTCACACACCAGCAGGTTA

TNF

F: AGGCACTCCCCCAAAAGATG

R: CCACTTGGTGGTTTGTGAGTG

IL10

F: GGTTGCCAAGCCTTATCGGA

R: GACACCTTGGTCTTGGAGCTTA

TGFB

F: ACTGGAGTTGTACGGCAGTG

R: GGGGCTGATCCCGTTGATTT

## Results

### RIG-I like receptor signaling pathway was screened as a significant risk factor for mortality in pneumonia patients

A total of 183 pneumonia patients were enrolled in the study, with a male-to-female ratio of 111:72 and an age range of 18–88 years. The 28-day mortality rate was 21.9% (40/183). Through GSVA, transcriptomic microarray data were converted into 185 signaling pathway datasets.

**Figure 1:**
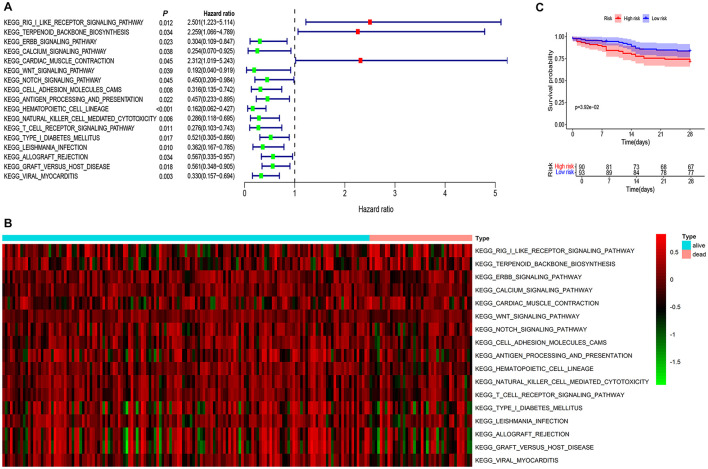
RIG-I like receptor pathway was screened as a significant risk factor for poor prognosis in pneumonia patients. **(A)** The combined analysis of GSVA and univariate COX regression identified 17 signaling pathways associated with the 28-day mortality of pneumonia patients, with a significance level of *P* < 0.05. Among these, the RIG-I Like receptor signaling pathway exhibited the highest hazard ratio of 2.501, with a 95% confidence interval of [1.223–5.114]. **(B)** A heatmap displayed the distribution of these 17 signaling pathways among the 183 pneumonia patients. **(C)** Survival analysis indicated that patients with high expression of the RIG-I Like receptor signaling pathway had a significantly higher mortality rate compared to the low-expression group (*P* = 0.039).

Univariate Cox analysis revealed that 17 signaling pathways were associated with the 28-day mortality of pneumonia patients, with a significance level of *P* < 0.05 (Figure 1A). A heatmap displayed the distribution of these 17 signaling pathways among the 183 pneumonia patients (Figure 1B). Among these, the RIG-I-like receptor signaling pathway exhibited the highest hazard ratio (HR) of 2.501, with a 95% confidence interval of [1.223–5.114]. Therefore, the RIG-I-like pathway is identified as the focal point of this research project.

Using the median value of the RIG-I Like receptor pathway data as the cutoff, pneumonia patients were categorized into high-risk and low-risk groups. Survival analysis indicated that patients with high expression of the RIG-I Like receptor pathway had a significantly higher mortality rate compared to the low-expression group (*P* = 0.039), shown in Figure 1C.

These results suggest that the RIG-I Like receptor signaling pathway may be a critical risk factor for mortality in pneumonia patients, and inhibiting this pathway may potentially reduce the mortality rate in these patients.

### RIG012 effectively inhibits RIG-I like receptor pathway in KP mouse

To investigate whether the inhibition of the RIG-I Like receptor pathway can improve pneumonia prognosis, we first established a pneumonia model in mice by intratracheally injecting KP. Subsequently, the small molecule compound RIG012, a specific inhibitor of the RIG-I signaling pathway, was administered via tail vein injection, while the control group received an equal volume of PBS.

**Figure 2:**
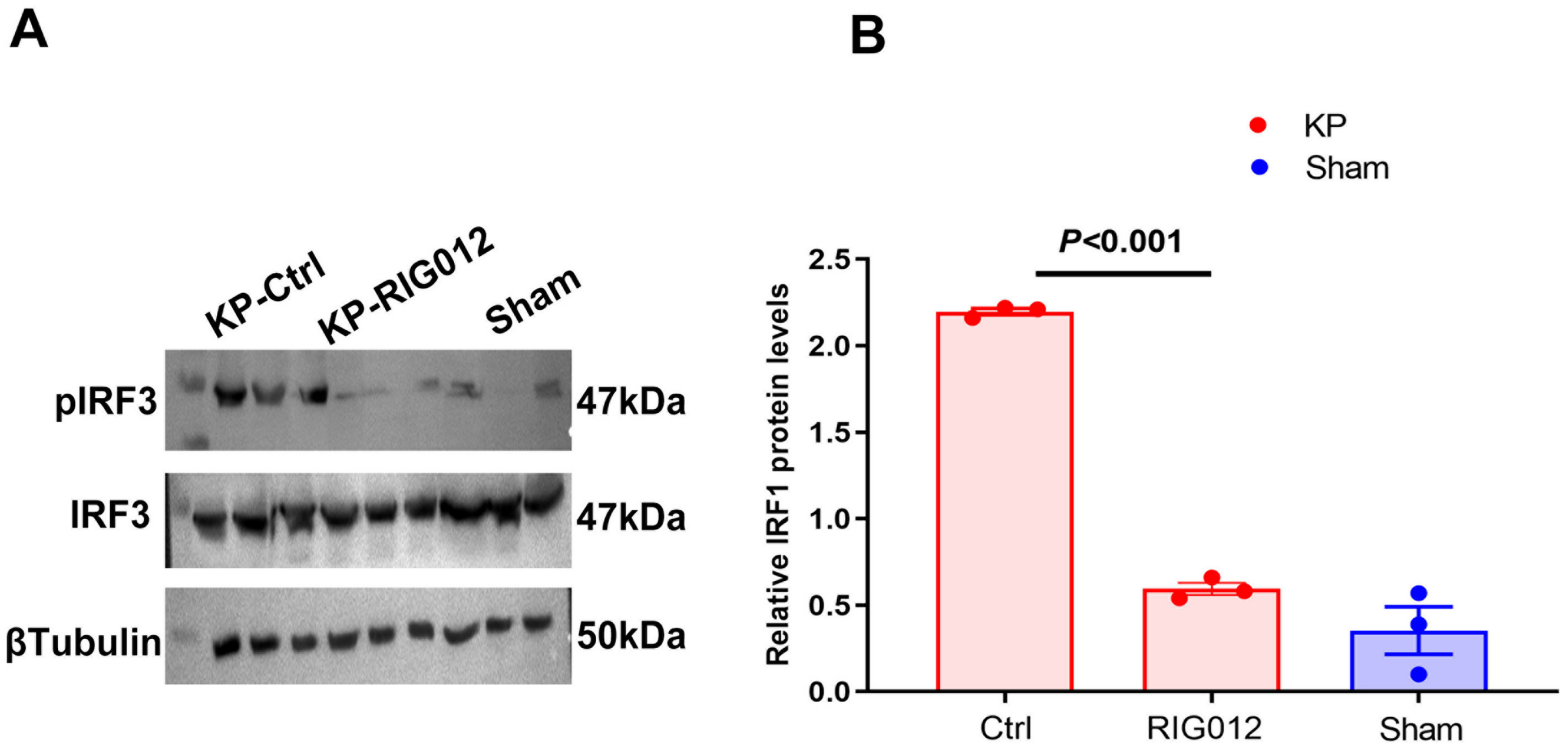
RIG012 effectively inhibits RIG-I like receptor signaling pathway in KP mouse. **(A, B)** Western blot analysis of lung tissue homogenates indicated that RIG012 effectively inhibited the activity of the RIG-I like pathway, *P* < 0.001.

Western blot analysis of lung tissue homogenates indicated that RIG012 effectively inhibited the activity of the RIG-I Like receptor pathway, with a significance level of *P* < 0.001 (Figure 2).

### RIG012 effectively improves lung injury in KP mice

**Figure 3:**
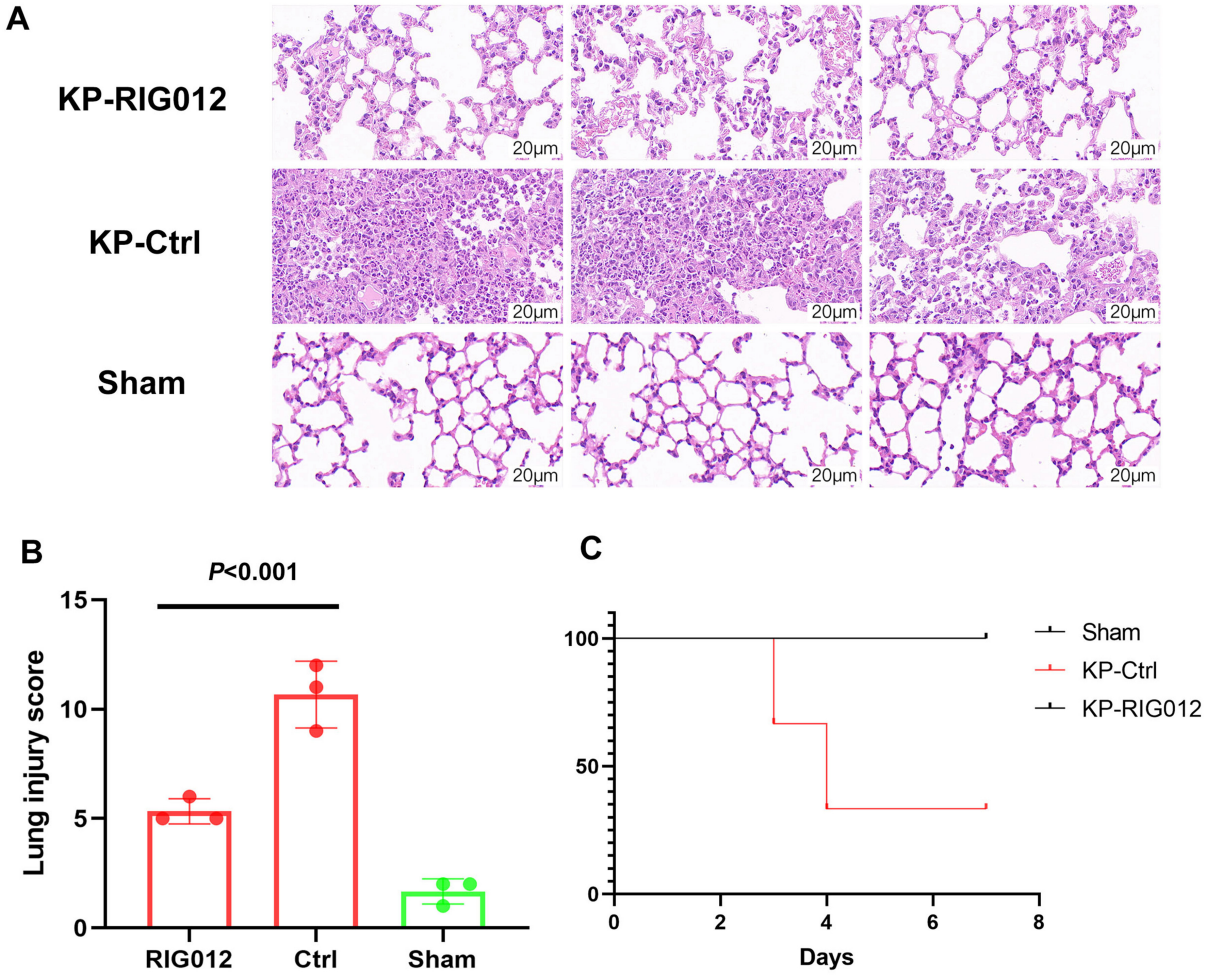
RIG012 effectively improves lung injury in KP mice. **(A)** H&E staining of lung tissues from mice in the RIG012 treatment group, treatment control group, and sham surgery group. **(B)** Lung injury scores were utilized to assess the protective effects of RIG012 on lung tissue. **(C)** Further survival analysis revealed that all mice in the RIG012 treatment group survived, while two mice in the control group (Ctrl) died within 7 days. KP, *Klebsiella pneumoniae*.

To evaluate whether RIG012 can enhance pneumonia prognosis, lung tissues from each group of mice were collected to prepare pathological sections. Lung injury scores were utilized to assess the protective effects of RIG012 on lung tissue. The results indicated that RIG012 significantly improved the lung injury scores in KP mice, with a significance level of *P* < 0.001 (Figure 3). Further survival analysis revealed that all mice in the RIG012 treatment group survived, while two mice in the control group (Ctrl) died within 7 days. These results suggest that RIG012 may potentially improve outcomes in pneumonia.

### RIG012 effectively reduces pulmonary inflammatory response

Recent studies have indicated that the RIG-I Like receptor signaling pathway is a significant mechanism contributing to inflammatory lung injury. We hypothesized that the mechanism through which RIG012 improves pneumonia prognosis in mice is by reducing pulmonary inflammatory responses. To validate this hypothesis, lung tissue homogenates from each group of mice were collected, and quantitative qPCR was employed to assess the expression levels of pro-inflammatory factors IL1B and TNF, as well as the anti-inflammatory factors IL10 and TGFB.

**Figure 4:**
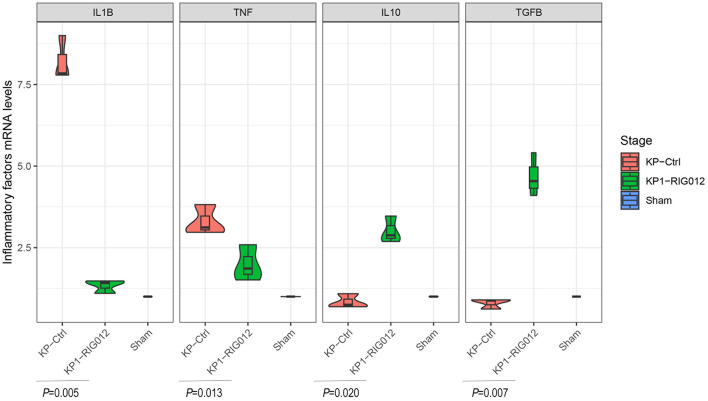
RIG012 effectively reduces pulmonary inflammatory response. The qPCR was employed to assess the expression levels of pro-inflammatory factors IL1B and TNF, as well as the anti-inflammatory factors IL10 and TGFB in lung tissue homogenates from each group of mice.

The results indicated that RIG012 significantly reduced the expression of pro-inflammatory factors in KP mice while increasing the levels of anti-inflammatory factors, *P* < 0.05 (Figure 4).

## Discussion

Pneumonia is a common clinical condition, with mortality rates as high as 40% in severe cases. The treatment of pneumonia primarily relies on antibiotics. However, if pneumonia progresses to acute respiratory distress syndrome and sepsis, leading to multiple organ dysfunction, necessary organ support therapies can be crucial in sustaining the lives of patients with severe pneumonia, providing a critical window for antibiotic efficacy. Nevertheless, there is currently a lack of adjunctive therapies targeting the pathophysiological mechanisms of pneumonia (1–4). Pneumonia can cause dysregulation of the immune system and may involve other molecular mechanisms within the body. Therefore, inhibiting harmful molecular pathways could potentially enhance the effectiveness of antibiotic therapy in treating pneumonia (13, 14). Severe pneumonia can lead to sepsis and acute respiratory distress syndrome (ARDS), further increasing mortality rates. This project focuses on patients with pneumonia complicated by sepsis, which constitutes a more severe form of pneumonia. The results indicate that RIG012 treatment may be effective in this context.

GSVA is a non-parametric, unsupervised analytical method primarily utilized for assessing gene set enrichment results in transcriptomic data from microarrays. This technique involves transforming the gene expression matrix across different samples into an expression matrix for gene sets, thereby evaluating whether specific metabolic pathways are enriched among the samples (7, 8). In simpler terms, GSVA converts the gene expression matrix (with gene names as row labels and sample names as column labels) into a pathway matrix (with pathway names as row labels and sample names as column labels). When combined with traditional statistical methods such as survival analysis, the GSVA algorithm can effectively uncover pathogenic mechanisms associated with disease prognosis.

The RIG-I Like receptor pathway is a crucial immune activation pathway in the human body. Initially, research suggested that this pathway was primarily involved in the antiviral defense mechanisms by facilitating the binding of RIG-I protein to viral RNA, resulting in the phosphorylation and nuclear translocation of the transcription factor IRF3, which in turn initiates the transcription of interferons. Recent studies have demonstrated that the RIG-I Like receptor pathway, in addition to mediating interferon production, also interacts with classic inflammatory signaling pathways such as NFκB, contributing to excessive inflammatory responses (15–20). In the context of pneumonia, the pathogenic microbes not only inflict direct damage to lung tissue but also mediate excessive inflammatory responses that can compromise the epithelial-endothelial barrier of the lungs. This may elucidate the mechanism by which GSVA analysis associates the RIG-I Like receptor pathway with poor prognosis in pneumonia patients.

Our study found that the RIG-I-like receptor pathway has the highest hazard ratio (HR) value of 2.501, with a wide confidence interval of [1.223–5.114]. This suggests that for each unit increase in the RIG-I-like pathway, the risk of mortality from pneumonia increases 2.5-fold. This is also the rationale for selecting the RIG-I-like pathway as the primary focus of our research. Additionally, the wide confidence interval of the HR indicates the presence of patient heterogeneity. Future studies that incorporate a larger sample size and conduct comprehensive subgroup analyses may be beneficial in exploring and elucidating this phenomenon.

RIG012 may serve as an effective adjunctive therapy for pneumonia. RIG012 is a small molecular compound that specifically targets RIG-I Like receptors, effectively inhibiting the RIG-I Like receptor pathway (21). Our animal studies have also demonstrated that RIG012 significantly suppresses the activity of this signaling pathway. Additionally, we observed that RIG012 can effectively mitigate lung damage caused by *Klebsiella pneumoniae* and shows a trend toward improving mortality rates in pneumonia-induced mice. Further experiments suggest that RIG012 may alleviate excessive pulmonary inflammation by inhibiting the RIG-I Like receptor pathway, thereby serving as an adjunctive treatment for lung damage resulting from pneumonia.

This study has certain limitations. First, the included mice were all male, and although previous research suggests that the RIG-I-like signaling pathway is not associated with sex, the issue of sex bias warrants attention. Second, the RIG012 treatment experiments were based on prior research and experimental data regarding dosage and duration; future studies should incorporate concentration and time gradient experiments. Finally, the databases utilized in this research are public and lack clinical information, such as the specific pathogens causing pneumonia. Future investigations should conduct larger-scale clinical studies to clarify the relationship between the RIG-I-like pathway and pneumonia prognosis, as well as to draw conclusions from subgroup analyses considering factors such as sex and pathogens.

## Conclusion

Through bioinformatics analysis and validation in animal experiments, we have discovered that RIG012 may represent a novel adjunctive therapeutic agent for pneumonia.

## Data Availability

The original contributions presented in the study are included in the article/Supplementary material, further inquiries can be directed to the corresponding authors.
